# Development and Validation of a Measure of Birth-Related PTSD for Fathers and Birth Partners: The City Birth Trauma Scale (Partner Version)

**DOI:** 10.3389/fpsyg.2021.596779

**Published:** 2021-03-03

**Authors:** Rebecca Webb, Ann M. Smith, Susan Ayers, Daniel B. Wright, Alexandra Thornton

**Affiliations:** ^1^Centre for Maternal and Child Health Research, School of Health Sciences, City, University of London, London, United Kingdom; ^2^Neonatal Intensive Care Unit, Homerton University Hospital NHS Foundation Trust, London, United Kingdom; ^3^Department of Educational Psychology and Higher Education, University of Nevada, Las Vegas, NV, United States; ^4^Perinatal Mental Health Service, West London NHS Trust, St Bernard’s Hospital, London, United Kingdom

**Keywords:** birth trauma, PTSD, fathers, partners, birth

## Abstract

Research suggests that some fathers and birth partners can experience post-traumatic stress disorder (PTSD) after witnessing a traumatic birth. Birth-related PTSD may impact on many aspects of fathers’ and birth partners’ life, including relationship breakdown, self-blame and reducing plans for future children. Despite the potential impact on birth partners’ lives there is currently no measure of birth-related PTSD validated for use with birth partners. The current study therefore adapted the City Birth Trauma Scale for use with birth partners. The City Birth Trauma Scale (Partner version) is a 29-item questionnaire developed to measure birth-related PTSD according to DSM-5 criteria: stressor criteria (A), symptoms of re-experiencing (B), avoidance (C), negative cognitions and mood (D), and hyperarousal (E), as well as duration of symptoms (F), significant distress or impairment (G), and exclusion criteria or other causes (H). A sample of 301 fathers/birth partners was recruited online and completed measures of birth-related PTSD, bonding, and demographic details. Results showed the City Birth Trauma Scale (Partner version) had good reliability (α = 0.94) and psychometric and construct validity. The fathers/birth partners version has the same two-factor structure as the original scale: (1) general symptoms and (2) birth-related symptoms, which accounted for 51% of the variance. PTSD symptoms were associated with preterm birth and maternal and infant complications. Overall, the City Birth Trauma Scale (Partner version) provides a promising measure of PTSD following childbirth that can be used in research and clinical practice.

## Introduction

Post-traumatic stress disorder (PTSD) is a trauma and stressor-related disorder that may develop following direct or indirect exposure to, or witnessing of, actual or threatened death, serious injury or sexual violence (American Psychiatric Association, 2014). Childbirth can act as a traumatic stressor in the development of postnatal PTSD (McKenzie-McHarg et al., 2015), and it is estimated that this affects approximately 4% of women. Prevalence increases to 18.5% in high risk samples, such as women who experienced emergency cesarean sections, severe fear of birth, a history of sexual/physical violence or childhood abuse, babies that were born preterm or very ill or women who had severe pregnancy complications (e.g., HELLP syndrome) (Dikmen Yildiz et al., 2017).

Approximately 97% of women will have their partner or another close friend or family member with them during birth (Care Quality Commission, 2020). Approximately 90% of fathers will attend the birth (Redshaw and Henderson, 2013), followed by a sister, mother, mother in law or female friend (Bohren et al., 2019). A meta-synthesis of the experience of birth partners found that some (both male and female) were deeply affected by witnessing a woman’s pain during labor, such as through feelings of frustration, fear, and helplessness (Bohren et al., 2019). This suggests being a birth partner may increase the risk of developing PTSD. In the context of childbirth, a father/birth partner witnessing a complicated birth involving his/her partner and/or child that has resulted in actual or threatened injury or death could qualify as fulfilling the DSM-5 Stressor A1 Criterion for PTSD. Rates of post-traumatic stress symptoms in birth partners have been found to vary in questionnaire studies from 0% to 8% of men attending their child’s birth, depending on the symptoms being measured (Ayers et al., 2007; Bradley et al., 2008). Further, research suggests that men do not always ask for support and there is a lack of recognition or comprehension regarding the issue by healthcare professionals (Hinton et al., 2014; Poh et al., 2014; White, 2014; Elmir and Schmied, 2016). These factors may lead to the psychological needs of fathers/birth partners not being recognized and subsequently fathers/birth partners not being provided with appropriate support or treatment. Case studies and qualitative research suggests some men may continue for years without seeking professional support for the effects of birth trauma (Beck et al., 2013; Hinton et al., 2014; White, 2014).

Research around risk factors for the development of PTSD after witnessing a traumatic birth has mainly focused on fathers, with the exception of Hinton et al. (2014) who interviewed one female partner. Risk factors include pregnancy and birth related factors such as witnessing near-miss obstetric emergencies or complicated births, e.g., emergency cesarean sections (Hinton et al., 2014; Elmir and Schmied, 2016); preterm birth (Stramrood et al., 2013) and complications during the birth (Ayers et al., 2007). Demographic risk factors include higher paternal age (Stramrood et al., 2013); fewer children, unplanned pregnancy (Bradley et al., 2008);and being a single father (Skari et al., 2002). Predisposing psychological factors include depression and trait anxiety (Iles et al., 2011; Zerach and Magal, 2016); and interpersonal risk factors include poor communication, support or treatment by healthcare professionals (Hinton et al., 2014; Poh et al., 2014; White, 2014; Elmir and Schmied, 2016).

The impact on the father/birth partner of birth-related PTSD has not been widely researched but qualitative and case studies suggest in fathers it is associated with social isolation, job loss, mental breakdown, vasectomy and sexual scarring (Hinton et al., 2014; White, 2014). For the family, PTSD may lead to relationship breakdown, financial insecurities and affect the parent–baby bond (Nicholls and Ayers, 2007; Hinton et al., 2014; White, 2014). Furthermore, a qualitative study of 10 men in the United Kingdom who had witnessed their partner’s labor and childbirth and perceived a threat to their partner or infant’s life or physical wellbeing found some men blamed themselves for the traumatic events during birth, and reported that the traumatic birth affected their parenting and plans for future children, as well as the couple’s relationship (Bristow, 2016).

Despite the potential impact of PTSD on fathers and birth partners, it remains under-researched. Lack of research evidence and awareness means it is also not recognized in maternity services. Routine assessment of fathers and birth partners’ mental health is neither conducted nor recommended in clinical guidelines (e.g., The National Institute for Health and Care Excellence, 2014). One of the barriers to routine assessment of fathers and birth partners is that there are no validated tools specifically for assessing birth-related PTSD with fathers and birth partners. To date, research has used four measures of PTSD symptoms after birth with fathers and birth partners: the Post-traumatic Stress Disorder Questionnaire (PTSD-Q); Perinatal PTSD Questionnaire (PPQ); Post-Traumatic Stress Disorder Diagnostic Scale (PDS); and the Impact of Events Scale (IES). The PTSD-Q (Czarnocka and Slade, 2000) is a self-report questionnaire adapted from a diagnostic interview and can be used to assess symptoms of posttraumatic stress (Watson et al., 1991). The PTSD-Q has been used in two studies of birth partners, however, it is not specifically designed to measure PTSD in response to childbirth (Bradley et al., 2008; Iles et al., 2011). The PPQ (Callahan and Borja, 2008) is a 14-item scale which measures perinatal PTSD and has been used in a few studies with fathers/birth partners (Pierrehumbert et al., 2003; Koliouli et al., 2016; Janis et al., 2017). However, this measure does not ask about traumatic stressor criteria or cover diagnostic criteria. The PDS (Foa et al., 1993) is a 49-item self-report measure that assesses all of the DSM-IV criteria for PTSD (American Psychiatric Association, 2000) and has been used in four studies with birth partners (Parfitt and Ayers, 2009; Stramrood et al., 2013; Horsch et al., 2017; Janis et al., 2017). However, again it is not specifically designed to measure postpartum PTSD. The IES (Horowitz et al., 1979) is a 15-item scale that measures symptoms of intrusions and avoidance. The IES is the most widely used measure with birth partners (Johnson, 2002; Skari et al., 2002; Ayers et al., 2007; Bradley et al., 2008; Gürber et al., 2017; Winter et al., 2018), however, it was not designed for use in the perinatal period. In addition, none of these scales measure the updated DSM-5 diagnostic criteria for PTSD. Thus, all four measures have limitations and none have been validated specifically for use with fathers and birth partners.

The City Birth Trauma Scale (Ayers et al., 2018) is a 29-item questionnaire developed to measure birth-related PTSD according to DSM-5 criteria (see Table 1). The original questionnaire was developed for use with postpartum women and has good reliability (Cronbach’s α = 0.92), psychometric validity and is easy to understand (Flesch reading score 64.17) (Ayers et al., 2018). Validations of translated versions of the City BiTS also find similar factor structure, reliability and validity (Handelzalts et al., 2018; Nakić Radoš et al., 2020; Caparros-Gonzalez et al., 2021). This is therefore a promising measure to be validated for use with fathers/birth partners. Therefore, to overcome the lack of validated measured available for assessing PTSD after birth in fathers/birth partners, the aim of the study was to adapt the City BiTS to create a partner version and examine its validity and reliability.

**TABLE 1:** DSM-V diagnostic criteria for PTSD.

Criterion	Description
A – Stressor criteria (one required)	A1: The person was exposed to: death, threatened death, actual or threatened serious injury, or actual or threatened sexual violence, in the following way(s):
	Direct exposure
	Witnessing the trauma
	Learning that a relative or close friend was exposed to a trauma
	Indirect exposure to aversive details of the trauma, usually in the course of professional duties (e.g., first responders, medics)
	A2: The person’s response involved intense fear, helplessness or horror
B – Symptoms of re-experience (one required)	The traumatic event is persistently re-experienced, in the following way(s):
	Intrusive thoughts
	Nightmares
	Flashbacks
	Emotional distress after exposure to traumatic reminders
	Physical reactivity after exposure to traumatic reminders
C – Avoidance (one required)	Avoidance of trauma-related stimuli after the trauma, in the following way(s):
	Trauma-related thoughts or feelings
	Trauma-related reminders
D –Negative cognitions and mood (two required)	Negative thoughts or feelings that began or worsened after the trauma, in the following way(s):
	Inability to recall key features of the trauma
	Overly negative thoughts and assumptions about oneself or the world
	Exaggerated blame of self or others for causing the trauma
	Negative affect
	Decreased interest in activities
	Feeling isolated
	Difficulty experiencing positive affect
E – Hyperarousal (two required)	Trauma-related arousal and reactivity that began or worsened after the trauma, in the following way(s):
	Irritability or aggression
	Risky or destructive behavior
	Hypervigilance
	Heightened startle reaction
	Difficulty concentrating
	Difficulty sleeping
F – Duration of symptoms (required)	Symptoms last for more than 1 month.
G – significant distress or impairment (required)	Symptoms create distress or functional impairment (e.g., social, occupational).
H - Exclusion criteria (required)	Symptoms are not due to medication, substance use, or other illness.

## Materials and Methods

### Design

A cross-sectional psychometric study to assess the reliability and validity of the City BiTS (Partner version) using responses to an online survey.

### Participants

Individuals were eligible for the study if they were present at their partner’s labor or birth within the past 5.5 years (a wide time frame was used to ensure we could recruit an adequate sample size); were 18 years and older; and had a good enough command of English to understand and respond to the questions.

A total of 383 eligible fathers/birth partners started the questionnaire, of these 301 (78.6%) fathers/birth partners were included in the final sample. Remaining participants (*n* = 82) were excluded because they dropped out before completing the questionnaire (*n* = 58, 15.4% of eligible participants), their child was over the age of 5.5 years (*n* = 14, 3.7% of eligible participants) or their infant’s date of birth was unreadable or incorrect (*n* = 10, 2.6% of eligible participants).

### Measure

The City BiTS was written to correspond directly with DSM-5 criteria but adapted to be specific to childbirth and contains 29 items. The City BiTS (Partner version) and scoring information is available online at https://blogs.city.ac.uk/citybirthtraumascale/. Criterion A (Q1–2) items are scored on a yes/no scale. DSM-5 symptoms Criteria B to E (Q3–22) are measured using 20 items that measure the frequency of symptoms for these items over the last week, rated on a four-point scale with scores from zero (not at all) to three (five or more times). The total symptom scores for Criteria B to E range from 0–60 and symptoms are considered present if an item is rated as one or more. Two questions (Q23–24), scored the same as Criterion B to E, identify a dissociative subtype so are not symptoms of PTSD. The scores for Criteria F (Q26) range from zero (symptoms < 1 month/no symptoms) to two (symptoms lasting > 3 months). Criterion G items (Q27–28) are rated as yes/sometimes/no and the exclusion criteria item (Q29) is rated as yes/maybe/no. The City BiTS can be scored to measure PTSD symptoms [including subscales, total PTSD symptoms, dissociative symptoms or symptom clusters as identified by previous research (Ayers et al., 2018; Handelzalts et al., 2018; Nakić Radoš et al., 2020; Caparros-Gonzalez et al., 2021)] or PTSD diagnoses.

The City BiTS was adapted for use with fathers and birth partners by altering the instructions slightly and ensuring questions for stressor criteria A were phrased to ask whether they thought their partner or baby might be seriously injured or die. Fathers/birth partners were classed as fulfilling Criteria F and G if they scored one or more on symptom duration and for distress or impairment, respectively. Fathers/birth partners who responded to the exclusion criteria question (Q29) as “yes” or “maybe” were considered not to have PTSD and therefore removed from estimates of PTSD prevalence.

To evaluate construct validity additional questions were asked which measured: (1) perceived trauma (“On a scale of 1–5 please comment on how traumatic you found the birth” [1 = not at all – 5 = extremely traumatic]); (2) maternal complications (“Did your partner suffer from any complications during the birth? [yes/no]. “If yes, what happened?” [Optional free text]); (3) infant complications (“Did your baby suffer from any complications during the birth?” [yes/no]. “If yes, what happened?” [Optional free text]); and (4) Father/parent – infant bond (“Since the birth, how bonded do you feel to your baby?” [1 = Extremely bonded – 5 = not at all bonded]).

### Procedure

This study was approved by City, University of London Research Governance Ethics Committee. The City BiTS (Partner version) and questions about participants demographic details were hosted on an online survey platform Qualtrics. The survey was available online for 9 months from June 2016 to March 2017. Participants were recruited though adverts placed on social media websites of United Kingdom perinatal charities (i.e., BLISS, PANDAS, DADS Matter UK, and the Birth Trauma Association), Twitter, and a study website created on Facebook. Consent to advertise the survey on charity websites was sought from moderators and research officers of relevant organizations and websites. Social media adverts contained hyperlinks to frequently asked questions and a link to the survey. Recruitment adverts were specifically targeted to fathers; however, the study did not ask participants for their sex or what their relationship was to the baby/child. It is therefore probable the sample was of fathers but possible that other types of birth partners were included. We therefore refer to fathers/birth partners throughout.

Participants were given information about the study and had to tick a box to confirm they were 18 years or older and consented to take part in the study. If participants did not tick this box they were unable to progress to the survey. Participation was voluntary and anonymous unless participants chose to give their email address to receive their assessment scores and/or a summary of the study results.

### Statistical Analysis

Analyses were conducted using the statistics environment R (R Core Team, 2019) and SPSS (IBM Corp, 2017). Descriptive statistics were calculated for all 22 items in the scale (see Supplementary Material). To assess symptom severity according to the DSM-5 criteria, the sums of subscales B–E were calculated to give scores for re-experiencing, avoidance, negative cognitions and mood, and hyperarousal. These were also totaled to provide a total PTSD symptoms score. Responses to stressor criteria were calculated, as were responses to questions about the onset of symptoms, duration, distress and impairment. The association between PTSD symptoms and time since birth was calculated using a one-way ANOVA. To test reliability, Cronbach’s alpha was calculated which estimates how well the set of questions measures a single overall construct. To test factorial validity an exploratory factor analysis was carried out as in Ayers et al. (2018). A two-factor solution with varimax rotation was sought. Additionally, a confirmatory factor analysis was carried out using two models. Two confirmatory models were examined. The first model is where each set of items B, C, D, and E, are influenced by separate factors, and these factors are correlated. The second is the two-factor solution as identified in Ayers et al. (2018). As an exploratory factor analysis was carried out it was not possible to do hypothesis tests for the confirmatory factor analysis. Therefore, BIC values were used to determine the fit of the models.

Further, the readability of the scale was determined using the Flesch readability scale and years of formal education required using the Gunning Fog index. As the data were not normally distributed, to test known-group validity, non-parametric Mann–Whitney *U*, and Kruskal–Wallis tests were carried out using birth-related symptoms, general symptoms and total symptoms as the dependent variables, and whether or not the mother or baby had any complications during the labor and birth (yes vs. no), what type of birth the mother had (unassisted vaginal vs. assisted vaginal or emergency CS), the partners bond with the baby, and the partners subjective rating of the birth as traumatic (1 = not traumatic – 5 = extremely traumatic) as the independent variables.

### Screening of Items

Initial data screening was conducted to examine whether any questions were not performing well. First, the range of each question was examined to ensure there were no restricted items where participants did not use the full range of the scale. This confirmed all questions used the full range. Secondly, the distributions of the questions were examined through inspection of skewness values and histograms. As with the original City BiTS scale, the majority of the symptom subscale items (B–E) were positively skewed (17 out of 20). PTSD scales are frequently skewed when used in normal populations (Ayers et al., 2018) and this was expected. Therefore, it was not appropriate to remove questions on this basis. Finally, questions were screened and noted if they were too highly correlated with other questions *r* > 0.90 (0 questions) or did not significantly correlate with other questions (0 questions). Eighty four percent of the correlations were between *r* = 0.20 and *r* = 0.60 (IQR: 0.31, 0.49). The highest correlation was *r* = 0.76 between Q6B (getting upset when reminded of the birth) and Q7B (feeling tense or anxious when reminded of the birth).

## Results

### Sample Characteristics

Fathers/birth partners included in the final sample (*n* = 301) ranged in age from 21 to 60 years (*M* = 34.59; *SD* = 6.08). From those that responded to the demographic questions, the majority of fathers/birth partners were first-time parents (*n* = 143; 54.20%) and most were White (*n* = 183; 92.00%). The children were aged from 0 to 64 months (*M* = 13.20; *SD* = 13.08) and most were born by unassisted vaginal birth (*n* = 85; 40.70%) or emergency cesarean birth (*n* = 83; 39.70%). See Table 2 for sample characteristics.

**TABLE 2:** Sample characteristics.

Characteristic	M (SD)
Fathers/Birth partners’ age (years; responses *n* = 249)	34.59 (6.08)
Children’s age (months; responses *n* = 301)	13.20 (13.08)
**Characteristic**	***N* (%)**
**Time since birth (responses *n* = 301)**	
Less than 6 months	97 (32.2)
6–12 months	110 (36.5)
13–24 months	43 (14.3)
More than 24 months	51 (16.9)
**Ethnicity (responses *n* = 199)**	
White	183 (92.0)
Asian	7 (3.5)
Mixed	5 (2.5)
Black	2 (1.0)
Other	2 (1.0)
**Job role (responses *n* = 264)**	
Professional	122 (46.2)
Managerial or technical	67 (25.4)
Skilled (non-manual)	16 (6.1)
Skilled (manual)	38 (14.4)
Partially skilled	5 (1.9)
Unemployed	2 (0.8)
Other	14 (5.3)
**Relationship status (responses *n* = 266)**	
Married	192 (72.2)
Cohabiting	62 (23.3)
In a relationship, but not cohabiting	4 (1.5)
Single Separated	3 (1.1) 4 (1.5)
Divorced	1 (0.4)
**Type of birth (responses *n* = 209)**	
Unassisted vaginal birth	85 (40.7)
Assisted vaginal birth	35 (11.6)
Emergency cesarean	83 (39.7)
Elective cesarean	6 (2.9)
**Maternal complications during labor and birth**	
**(responses *n* = 300)**	
Yes	156 (52.0)
**Infant complications during labor and birth**	
**(responses *n* = 300)**	
Yes	169 (56.3)
**Father/Birth partner’s perception of bond with infant**	
**(responses *n* = 266)**	
Extremely bonded	143 (53.8)
Very bonded	61 (22.9)
Moderately bonded	45 (16.9)
Slightly bonded	13 (4.9)
Not at all bonded	4 (1.5)

There was a high proportion of preterm birth, maternal and infant complications. The majority of fathers/birth partners had a preterm infant (59.6%, *n* = 161) with the mean gestational age of 33.67 weeks (SD = 5.61; range = 21–43 weeks). Similarly, over half the sample reported maternal complications during birth (52%; *n* = 156) and/or infant complications during birth 56.3% (*n* = 169). Optional open text responses describing the complications experienced (*n* = 149) suggested the most common maternal complications were issues with cord/placenta [e.g., retained placenta, placental abruption, cord prolapse (*n* = 23); post-partum hemorrhage (*n* = 37); pre-eclampsia or issues with blood pressure (*n* = 24); and tears/episiotomies (*n* = 14)]. With regards to infants, the most commonly reported complications were the infant being born unable to breathe and needing resuscitation (*n* = 55); and pre-term birth (*n* = 49). Four fathers/birth partners reported that their infant died.

### PTSD Symptoms

The sum of the 20 PTSD symptom subscale items (scales B–E) has a possible range from 0 to 60 and the observed range was 0 to 54 (*M* = 17.38; *SD* = 13.89; IQR: 5.00–26.00). The majority of fathers/birth partners reported at least one symptom (*n* = 249, 82.7%). The distribution was positively skewed (skew = 0.75).

Responses to the stressor criteria A can be found in Table 3. It can be seen that 58% of fathers/birth partners thought their partner or baby would be seriously injured, and 52% thought their partner or baby would die during the birth. Stressor criteria A for a traumatic birth were fulfilled by 64% of the sample. Seventy-seven fathers/birth partners (26%, 95% CI: 21, 31) fulfilled all DSM-5 diagnostic criteria for PTSD. Twenty-one fathers/birth partners (7%) indicated their symptoms might be due to medication, alcohol, drugs or physical illness so excluding these fathers/birth partners meant 22% of the sample met diagnostic criteria for PTSD (95% CI: 18, 27). Time since birth was not associated with PTSD symptom severity [*F*(49,229) = 1.284, *p* = 0.115].

**TABLE 3:** PTSD and stressor criteria (*n* = 301).

Criteria	Yes Freq (%)	No Freq (%)
During the birth did you believe your partner or your baby would be seriously injured?	174 (58)	124 (42)
Did you believe your partner or your baby would die?	157 (52)	144 (48)
Criterion A (stressors)	193 (64)	108 (36)
Criterion B (re-experiencing)	212 (70)	89 (30)
Criterion C (avoidance)	147 (49)	154 (51)
Criterion D (negative cognitions and mood)	187 (62)	114 (38)
Criterion E (hyperarousal)	214 (71)	87 (29)
Criterion F (duration)	247 (82)	54 (18)
Criterion G (distress or impairment)	158 (52)	143 (48)
DSM-5 PTSD	77 (26)	224 (74)
Could these symptoms be due to medication, alcohol, drugs or physical illness?	21 (7)	280 (93)
DSM-5 PTSD after removing partners who meet potential exclusion criteria	66 (22)	235 (78)

Responses to questions about the onset of symptoms, duration, distress, and impairment are shown in Table 4. Not applicable responses are shown for the first two items, and then the percentages without these. This shows the majority of fathers/birth partners with symptoms reported onset within the first 6 months after birth (59%); and that their symptoms had lasted 3 months or more (45%). However, a proportion of fathers/birth partners reported their symptoms started before the birth (11%), suggesting they had pre-existing PTSD or related symptoms. There was an association between the onset of symptoms before vs. after birth and severity of symptoms, in that more fathers/birth partners reported feeling distressed if their symptoms started more than 6 months after birth (*n* = 9, 40.9%), rather than before birth (*n* = 9, 30%) or within the first 6 months after birth (*n* = 47; 28.3%), [χ^2^(2) = 6.50; *p* = 0.039; *V* = 0.186].

**TABLE 4:** Onset and impact of PTSD in Fathers/Birth partners.

Item	Response scale	*N* (%)	95% CI
Onset of symptoms	Before the birth	30 (11)	8–15
	First 6 months after birth	166 (59)	54–65
	More than 6 months after birth	22 (8)	5–12
	Not applicable	61 (22)	17–27
Duration of symptoms	Less than 1 month	54 (18)	14–23
	1–3 months	53 (18)	14–22
	More than 3 months	136 (45)	40–51
	Not applicable	58 (19)	15–24
Do these symptoms cause you a lot of distress?	Yes	66 (22)	18–27
	Maybe	78 (26)	21–31
	No	157 (52)	47–58
Do they prevent you from doing things you usually do?	Yes	42 (14)	10–18
	Maybe	60 (20)	16–25
	No	199 (66)	61–71
Could these symptoms be due to medication, alcohol, drugs or physical illness?	Yes	5 (2)	1–4
	Maybe	16 (5)	3–8
	No	280 (93)	90–95

### Factorial Validity

Statistical checks confirmed the sample was adequate for factor analysis (Kaiser-Rice MSA measure = 0.926) and correlations between questions were suitably large [Bartlett’s test of sphericity, χ^2^(190) = 3745.14, *p* < 0.001]. The exploratory factor analysis which sought two-factor solution with varimax rotation (Ayers et al., 2018) was found, and accounted for 50.81% of the variance. After rotation the first factor, which reflected general symptoms, accounted for 25.45% of the variance and the second factor, which reflected birth-related symptoms, accounted for 25.36% of the variance. Table 5 shows the item loadings and it can be seen that items loaded onto the same factors found in the women’s version (Ayers et al., 2018).

**TABLE 5:** Factor structure of the City BiTS (Partner version).

			Factor analysis of all symptoms (varimax rotation)	
		Uniqueness	General symptoms	Birth-related symptoms
**B**	**Intrusions**			
3	Recurrent or unwanted memories of the birth that you can’t control	0.36	0.26	0.75
4	Bad dreams or nightmares about the birth	0.69	0.25	0.49
5	Flashbacks to the birth and/or reliving the experience	0.50	0.20	0.68
6	Getting upset when reminded of the birth	0.30	0.23	0.80
7	Feeling tense or anxious when reminded of the birth	0.32	0.28	0.77
**C**	**Avoidance**			
8	Trying to avoid thinking about the birth	0.34	0.24	0.77
9	Trying to avoid things that remind me of the birth	0.45	0.22	0.71
**D**	**Negative mood and cognitions**			
10	Not being able to remember details of the birth	0.93	0.24	0.11
11	Blaming myself or others for what happened during the birth	0.67	0.34	0.46
12	Feeling strong negative emotions about the birth	0.36	0.42	0.68
13	Feeling negative about myself or thinking something awful will happen	0.50	0.62	0.34
14	Lost interest in activities that were important to me	0.39	0.72	0.30
15	Feeling detached from other people	0.30	0.77	0.32
16	Not being able to feel positive emotions	0.40	0.71	0.24
**E**	**Hyperarousal**			
17	Feeling irritable or aggressive	0.47	0.69	0.24
18	Feeling self-destructive or acting recklessly	0.61	0.57	0.25
19	Feeling tense and on edge	0.36	0.77	0.22
20	Feeling jumpy or easily startled	0.61	0.56	0.28
21	Problems concentrating	0.41	0.75	0.16
22	Not sleeping well because of things that aren’t due to the baby’s sleep pattern	0.58	0.61	0.21
	Total variance explained		25.45%	25.36%

*Items 23 and 24 do not measure symptoms of PTSD but identify a dissociative PTSD subtype so were therefore excluded from this analysis.*

Confirmatory factor analysis showed that both the four-factor (model 1, AIC = 13484; BIC = 13647) and two-factor (model 2, AIC = 13505; BIC = 13649) fit similarly (see Supplementary Material for more details).

### Reliability

The symptom subscales and total symptoms had good internal consistency with Cronbach’s alphas above the acceptable level of 0.7 (α = 0.94 for the total scale; range for subscales = 0.78–0.87; see Table 6). Removing items from the total scale, re-experiencing, avoidance, or hyperarousal subscales did not improve the Cronbach’s alpha. Cronbach’s alpha was high for the birth-related symptoms factor of the scale (α = 0.92) and for the general symptoms factor (α = 0.91).

**TABLE 6:** Cronbach’s alpha for total scale and subscales.

Scale	Number of items	α	95% CI
A: Stressor	2	0.78	0.74–0.81
B: Re-experiencing	5	0.87	0.85–0.89
C: Avoidance	2	0.82	0.79–0.85
D: Negative cognitions and mood	7	0.86	0.84–0.89
E: Hyperarousal	6	0.87	0.84–0.89
Total symptoms (without A)	20	0.94	0.93–0.95
Total scale	22	0.94	0.93–0.95

### Readability

The Flesch readability scale indicated that the City BiTS (Partner version) has a reading ease score of 57.8 meaning it should be easily understood by age 16–17 years. The number of years of formal education required to easily read the scale (Gunning Fog index) was 12.9.

### Known-Group Validity

Results of known groups analyses are shown in Table 7. This shows that fathers/birth partners who met criteria for a traumatic birth reported significantly more general symptoms (Mean rank = 81.34, Mann–Whitney *U* 7238, *p* < 0.001) and birth-related symptoms (Mean rank = 70.77, Mann–Whitney *U* 8060.5, *p* < 0.001). Fathers/birth partners who reported maternal complications during birth reported significantly more birth-related symptoms (Mean rank = 159.11; Mann–Whitney *U* 8178, *p* = 0.002) but not general symptoms (Mean rank = 143.54; Mann–Whitney *U* 9034, *p* = 0.373). Fathers/birth partners who reported infant complications reported significantly more birth-related symptoms (Mean rank = 162.91; Mann–Whitney *U* 7186, *p* < 0.001) and general symptoms (Mean rank = 156.70; Mann–Whitney *U* 6688, *p* < 0.001). Subjective rating as the birth as traumatic was associated with general symptoms (*rho* 0.45, *p* ≤ 0.001) and birth-related symptoms (*rho* 0.59, *p* < 0.001). General symptoms were also associated with poorer bonding with the baby (*rho* 0.21, *p* < 0.001).

**TABLE 7:** Known group analyses.

		Total PTSD symptoms		General PTSD symptoms		Birth-related PTSD symptoms	
		*Mdn* (*IQR*)	*Kruskal–Wallis*, *p*	*Mdn* (*IQR*)	*Kruskal–Wallis*, *p*	*Mdn* (*IQR*)	*Kruskal–Wallis, p*
Type of birth	Vaginal	13.5 (16.75)	3.84, 0.147	8 (11.00)	2.49, 0.288	4 (9.75)	5.36, 0.069
	Assisted vaginal	10 (20.00)		6 (11.00)		2 (9.00)	
	Emergency cesarean	15 (23.00)		9 (14.00)		5 (12.00)	
			***U, p***		***U, p***		***U, p***
Traumatic birth (criterion A)	Yes	20 (21.00)	7841.5, <0.001	11 (14.50)	7238, <0.001	8 (12.00)	8060.5, <0.001
	No	8 (12.25)		6 (8.50)		1 (4.00)	
Maternal complications	Yes	16 (24.25)	8222, 0.044	10 (17.00)	9034, 0.373	6 (11.00)	8178, 0.002
	No	13 (20.00)		9 (11.00)		3 (10.00)	
Infant complications	Yes	18 (23.00)	6322, <0.001	11 (13.00)	6688, <0.001	8 (12.00)	7186, <0.001
	No	10.5 (18.25)		6.5 (11.00)		3 (8.00)	
			***Rho, p***		***Rho, p***		***Rho, p***
Subjective rating of birth as traumatic	Not at all	1 (4.00)	0.559, <0.001	1 (4.00)	0.446, <0.001	0 (0.00)	
	Slightly traumatic	6.5 (11.75)		5.5 (9.00)		1 (2.75)	
	Moderately traumatic	10 (14.00)		6 (11.00)		3 (7.00)	0.587, <0.001
	Very traumatic	20 (17.00)		11 (9.50)		8 (10.00)	
	Extremely traumatic	30 (24.50)		17 (12.50)		12 (11.00)	
Bond with the baby	Extremely bonded	12 (25.00)	0.179, 0.003	7 (15.00)	0.211, 0.001	4 (12.00)	0.106, 0.086
	Very bonded	14 (20.50)		9 (13.50)		4 (10.00)	
	Moderately bonded	21 (15.00)		12 (9.50)		9 (9.50)	
	Slightly bonded	18 (26.00)		12 (15.40)		8 (13.00)	
	Not at all bonded	26^α^		15^α^		11^α^	

*^α^Not enough data to calculate interquartile range (*n* = 3). NB. Due to missing data *n* varies: traumatic birth (Criterion A) *n* = 207; type of birth *n* = 203; maternal and infant complications *n* = 277, subjective rating of birth and bond with baby *n* = 266.*

## Discussion

The aim of this paper was to adapt the City BiTS to create a version for fathers and birth partners and test the reliability and psychometric validity in a sample of fathers/birth partners. The results suggest the City BiTS (Partner version) has good reliability, is relatively easy to use, and has the same two-factor structure as the original City BiTS. Results showed 26% of fathers/birth partners in this sample met criteria for PTSD after birth on this scale, which reduced to 22% after excluding those whose symptoms could have been due to medication, alcohol, drugs or physical illness. Twenty-two percent of fathers/birth partners reported that their symptoms caused them a lot of distress.

### Psychometric Properties of the City BiTS

This study adds to the evidence that the City BiTS has good internal consistency. Internal consistency in this study (α = 0.94) is similar to that found in the original City BiTS scale and translated into Croatian (α = 0.90–0.92) (Ayers et al., 2018; Handelzalts et al., 2018; Nakić Radoš et al., 2020). This suggests the City BiTS (original) and City BiTS (Partner version) are reliable measures for assessing postpartum PTSD. However, further research is needed looking at other measures of reliability, such as test–retest.

The City BiTS (Partner version) also appears to have good validity. Known-group validity showed scores on the City BiTS were associated with partners subjective ratings of birth as traumatic and reporting complications with their partner or baby during the birth. Total and general symptoms were also associated with poorer parent-child bond.

The two-factor structure of the Partner version is the same as that found in psychometric studies of the original scale (Ayers et al., 2018; Handelzalts et al., 2018; Nakić Radoš et al., 2020). As with the original scale the item ‘*Not being able to remember details about the birth’* did not load strongly on either factor suggesting this item has consistently poor loadings in women and men after birth (Ayers et al., 2018; Handelzalts et al., 2018; Nakić Radoš et al., 2020; Caparros-Gonzalez et al., 2021). This may be because the birth of one’s baby is a central life event which is likely to be remembered in detail. This study also adds to the evidence that postpartum PTSD symptoms measured by the City BiTS are consistently explained by two factors, one relating to general symptoms and the other relating to birth-related symptoms (Ayers et al., 2018; Handelzalts et al., 2018; Nakić Radoš et al., 2020; Caparros-Gonzalez et al., 2021). Research examining the factor structure of postpartum PTSD using other measures also finds two factors that are broadly consistent with the City BiTS. For example, Ayers et al. (2009), Stramrood et al. (2010), and Reichenheim et al. (2018) identified two clusters of re-experiencing and avoidance and numbing and (hyper)arousal. However, not all studies of postpartum PTSD find this two-factor structure, Olde et al. (2006) identified a three-factor structure of intrusion, avoidance and hyperarousal, and a systematic review of PTSD symptoms’ latent structure in other populations even suggests six [e.g., Anhedonia model (Liu et al., 2014)^1^ or seven-factor (Hybrid model, Armour et al., 2015)^2^ models (Armour et al., 2016)].

The amount of postpartum PTSD variance explained by these two factors is over 50% in the Partner version and the original City BiTS (Ayers et al., 2018). However, a much lower proportion of the variance was explained by birth-related symptoms in the Partner version compared to the original scale for women [25% compared to 40–45%, respectively (Ayers et al., 2018; Handelzalts et al., 2018; Nakić Radoš et al., 2020)]. This suggests that birth-related symptoms and cognitions may be more influential in the development of PTSD in women than they are for fathers/birth partners.

It is interesting that the factor structure and item loadings for postpartum PTSD are the same in birth partners and women who went through labor and birth, despite potential differences in the way birth trauma may be experienced fathers/birth partners. For example, for women giving birth the threat is nearly always direct (i.e., to themselves or their baby *in utero*) and they will experience the physiological phenomena of labor and birth, which includes pain and invasive bodily procedures. In contrast, for fathers/birth partners the threat is indirect (i.e., to their partner and/or baby), they do not have the same physiological phenomena to cope with, but may feel ill-equipped and powerless to help. This is consistent with evidence that many fathers report feeling helpless during the birth of their child (Leonard, 1977; Nichols, 1993; Johansson et al., 2015; Elmir and Schmied, 2016) and often hide their emotions and feelings during labor to avoid upsetting their partners (Chandler and Field, 1997). A meta-ethnographic synthesis of qualitative studies of fathers’ experiences of complicated births found themes of ‘*the unfolding crisis*’ and ‘*stripped of my role: powerless and helpless.*’ Remaining themes were ‘*craving information*,’ and ‘*scarring the relationship*’ (Elmir and Schmied, 2016). Further research examining both women giving birth and fathers/birth partners experiences of birth trauma is therefore needed to increase understanding of possible differences between experienced and witnessed trauma, as well as between genders, in how trauma is experienced and expressed.

### Prevalence of PTSD

Although this study was not designed to establish prevalence, results suggested 26% of fathers/birth partners in our sample fulfilled diagnostic criteria for PTSD which is higher than the 7% of women with PTSD in the original City BiTS evaluation (Ayers et al., 2018) and the 3–4% prevalence of postpartum PTSD in women estimated by reviews and meta-analyses (Grekin and O’Hara, 2014; Dikmen Yildiz et al., 2017). Higher prevalence rates of 15–18% are observed in high risk samples, and as this sample was self-selected it is probable we recruited a high-risk sample of fathers/birth partners. Online sampling and recruitment through organizations such as the Birth Trauma Association and BLISS, a preterm birth charity, probably meant fathers/birth partners with PTSD were over-represented and we inadvertently recruited a high-risk sample. The high proportion of fathers/birth partners who believed that their partner or baby was going to be seriously injured (54%) or die (48%) is consistent with this, as is the number of infants born prematurely (59%), and the number of fathers/birth partners reporting complications with their partner or baby (52 and 56%, respectively). In the original City BiTS study these rates were much lower (15% and 14%, respectively) (Ayers et al., 2018).

### Limitations

A number of limitations should be considered before drawing conclusions. First, the sample in this study was recruited online so is self-selected and not representative. As discussed, prevalence rates are therefore unlikely to be representative and this study probably recruited high-risk fathers/birth partners who were more likely to have PTSD symptoms. This is reflected in the average preterm gestation of the infants when they were born and the number of fathers/birth partners who reported complications during birth for their partner and/or baby. Recruitment was targeted at fathers but sex of participants or their relationship to the baby/child was not recorded so it is possible our sample included birth partners who were not biological fathers or men. As research that looks at the experience of birth from the perspective of birth partners is usually carried out with fathers (Hinton et al., 2014; White, 2014; Bristow, 2016), it is not known whether birth partners or fathers would respond differently to the scale. This needs to be examined in future studies to ensure other types of birth partners are represented.

Demographic characteristics indicate the sample was predominantly white, which is similar to the United Kingdom population but results will not be representative of those from minority ethnic communities. Additionally, this study did not assess convergent or predictive validity. The original scale has high convergent validity with the Impact of Events Scale-Revised, Edinburgh Postnatal Depression Scale and Pittsburgh Sleep Quality Index (Handelzalts et al., 2018). More research is needed to establish convergent and predictive validity of the original scale and the Partner version; along with factor structure and reliability of the Partner version in more diverse samples of fathers/birth partners.

## Conclusion

This study is the first to adapt a specific measure of postpartum PTSD according to DSM-5 criteria for use with fathers and birth partners and examine its reliability and psychometric validity. Results suggest the City BiTS (Partner version) has good reliability, reasonable face validity and the same two-factor structure as the original scale. However, more research is needed to replicate and extend this study. In particular to examine PTSD symptoms, prevalence, and factor structure in more representative samples, as well as the psychometric properties of the City BiTS (Partner version) in more diverse groups of fathers/birth partners. Potential differences in how birth trauma is experienced and expressed by fathers compared to other birth partners needs to be explored. Future research should also include additional measures of reliability (e.g., test–retest) and validity (e.g., convergent, predictive) to establish the utility of the scale for research and clinical practice.

## Data Availability Statement

The raw data supporting the conclusions of this article will be made available by the authors, without undue reservation.

## Ethics Statement

The studies involving human participants were reviewed and approved by City, University of London Research Governance Ethics Committee. The patients/participants provided their written informed consent to participate in this study.

## Author Contributions

RW prepared the data for analysis, interpreted the data, and wrote the manuscript and manuscript revisions. AS carried out data collection and provided feedback on the manuscript. SA co-created the measure, overviewed the entire research process, provided feedback, and edited the manuscript. DW co-created the measure, carried out the analysis, and provided feedback on the manuscript. AT co-created the measure and provided feedback on the manuscript. All authors contributed to the article and approved the submitted version.

## Conflict of Interest

The authors declare that the research was conducted in the absence of any commercial or financial relationships that could be construed as a potential conflict of interest.

## Footnotes

1 Intrusion, avoidance, negative affect, anhedonia, anxious hyperarousal, and dysphoric arousal.

2 Re-experiencing, avoidance, negative affect, anhedonia, externalizing behaviors, anxious arousal, and dysphoric arousal.
