# Supplementary material for: Development and Validation of a Measure of Birth-Related PTSD for Fathers and Birth Partners: The City Birth Trauma Scale (Partner Version)
Source: Front Psychol. 2021 Mar 3;12:596779. doi: 10.3389/fpsyg.2021.596779 (PMC7966709; doi:10.3389/fpsyg.2021.596779)
Supplement: Supplementary file 1 [file Data_Sheet_1.PDF]

The City Birth Trauma Scale
(Partner Version)
Supplementary materials

Contents

1. Histograms and skewness..P.2
2. Inter-item correlations..P.3
3. Descriptive statistics for each scale item.......................................P.4
4. Reliability - Cronbach's alpha...P.5
5. Confirmatory factor analysis...P.6

1. Histograms and Skewness for all items

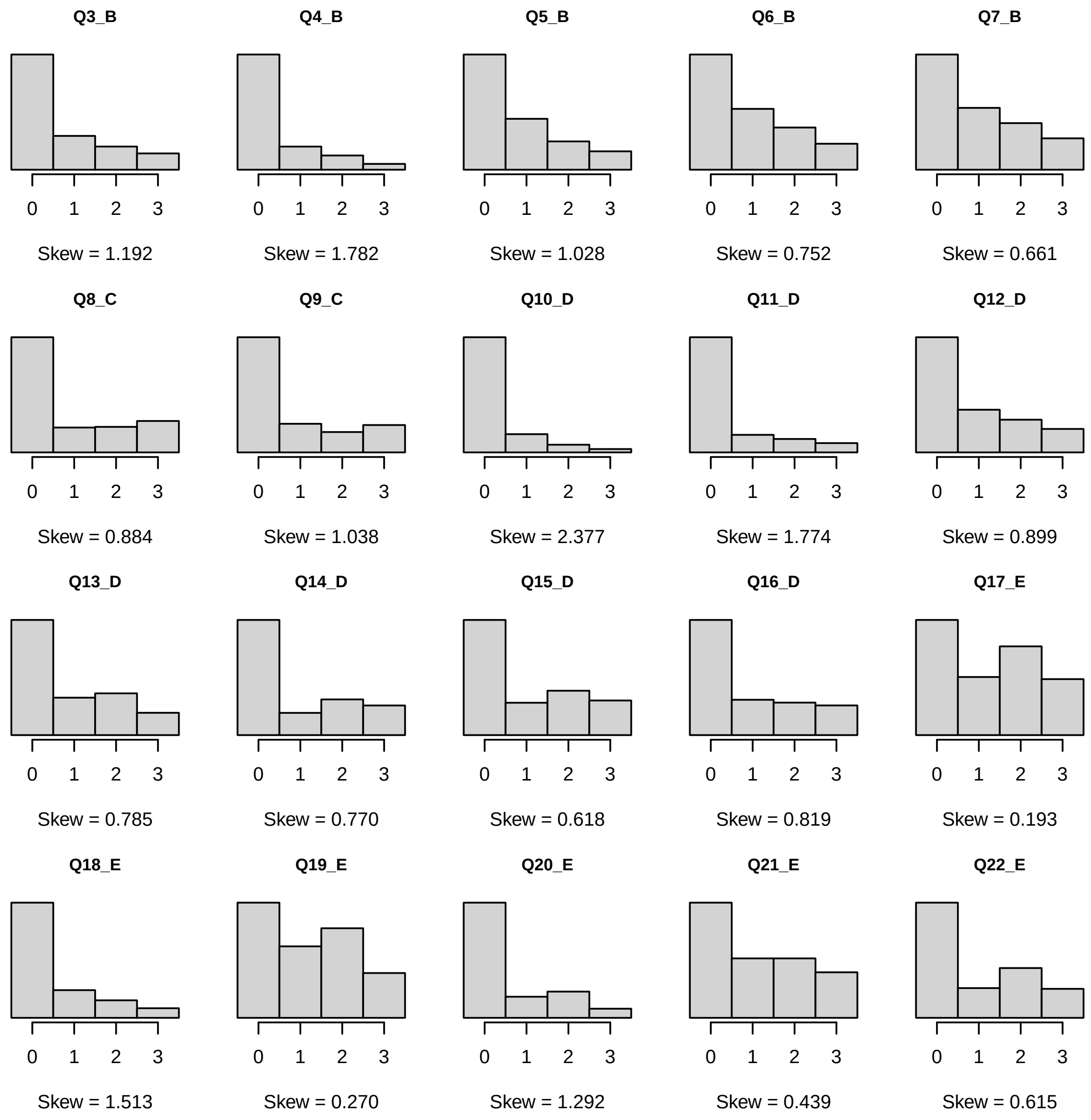

2. Inter-Item Correlations

	Q1_A	Q2_A	Q3_B	Q4_B	Q5_B	Q6_B	Q7_B	Q8_C	Q9_C	Q10_D	Q11_D	Q12_D	Q13_D	Q14_D	Q15_D	Q16_D	Q17_E	Q18_E	Q19_E	Q20_E	Q21_E
Q2_A	.64																				
Q3_B	.38	.40																			
Q4_B	.27	.29	.59																		
Q5_B	.30	.32	.69	.48																	
Q6_B	.38	.34	.63	.49	.60																
Q7_B	.41	.38	.62	.42	.54	.73															
Q8_C	.30	.34	.64	.33	.50	.65	.71														
Q9_C	.26	.26	.57	.38	.50	.60	.59	.69													
Q10_D	.10	-.01	.08	.08	.17	.10	.09	.18	.17												
Q11_D	.27	.24	.46	.39	.43	.43	.39	.36	.39	.24											
Q12_D	.38	.37	.59	.32	.53	.66	.64	.67	.56	.26	.56										
Q13_D	.37	.35	.37	.36	.37	.41	.42	.39	.38	.20	.46	.54									
Q14_D	.27	.30	.42	.36	.32	.40	.44	.38	.39	.16	.41	.50	.61								
Q15_D	.29	.32	.48	.32	.34	.42	.49	.48	.40	.24	.37	.53	.59	.76							
Q16_D	.25	.32	.41	.27	.30	.42	.45	.46	.39	.19	.34	.55	.52	.66	.74						
Q17_E	.29	.28	.39	.24	.32	.35	.37	.36	.35	.14	.32	.46	.48	.53	.57	.53					
Q18_E	.28	.28	.32	.29	.25	.37	.37	.32	.24	.08	.32	.42	.45	.43	.49	.49	.55				
Q19_E	.31	.34	.38	.27	.31	.37	.41	.38	.30	.26	.30	.47	.52	.54	.62	.57	.71	.54			
Q20_E	.31	.28	.36	.37	.37	.35	.39	.31	.31	.20	.26	.40	.41	.41	.51	.44	.44	.51	.54		
Q21_E	.19	.27	.31	.32	.30	.31	.33	.28	.27	.22	.36	.40	.53	.58	.59	.56	.53	.40	.66	.49	
Q22_E	.23	.24	.30	.38	.32	.31	.33	.28	.30	.13	.37	.36	.44	.48	.50	.46	.46	.36	.57	.44	.58

Table 1: Item correlations.

3. Descriptive Statistics for scale items

	N	F0	F1	F2	F3	mean
Q1_A	301	0.42	0.58			0.58
Q2_A	301	0.48	0.52			0.52
Q3_B	301	0.61	0.18	0.12	0.09	0.68
Q4_B	301	0.73	0.15	0.09	0.04	0.44
Q5_B	301	0.54	0.24	0.13	0.09	0.76
Q6_B	301	0.47	0.25	0.17	0.11	0.91
Q7_B	301	0.45	0.24	0.18	0.12	0.98
Q8_C	301	0.58	0.13	0.13	0.16	0.86
Q9_C	301	0.60	0.15	0.11	0.14	0.79
Q10_D	301	0.80	0.13	0.05	0.02	0.30
Q11_D	301	0.74	0.11	0.09	0.06	0.47
Q12_D	301	0.54	0.20	0.15	0.11	0.83
Q13_D	301	0.53	0.17	0.19	0.10	0.87
Q14_D	301	0.57	0.11	0.18	0.15	0.90
Q15_D	301	0.51	0.14	0.20	0.15	0.99
Q16_D	301	0.54	0.17	0.15	0.14	0.89
Q17_E	301	0.36	0.18	0.28	0.18	1.27
Q18_E	301	0.68	0.16	0.10	0.06	0.54
Q19_E	301	0.36	0.22	0.28	0.14	1.20
Q20_E	301	0.67	0.12	0.15	0.05	0.59
Q21_E	301	0.41	0.21	0.21	0.16	1.13
Q22_E	301	0.51	0.13	0.22	0.13	0.97

N = Valid cases, F0-F3 = the proportion of valid responses for values 0-3

4. Reliability - Cronbach's Alpha

Item	A	B	C	D	E	A–E	B–E
Q1_A	.636					.937	
Q2_A	.405					.936	
Q3_B		.828				.933	.933
Q4_B		.875				.935	.935
Q5_B		.848				.934	.934
Q6_B		.833				.933	.932
Q7_B		.847				.933	.932
Q8_C			.695			.933	.933
Q9_C			.483			.934	.934
Q10_D				.879		.939	.939
Q11_D				.855		.935	.935
Q12_D				.836		.932	.931
Q13_D				.835		.933	.933
Q14_D				.826		.933	.932
Q15_D				.820		.932	.931
Q16_D				.830		.933	.932
Q17_E					.839	.934	.933
Q18_E					.856	.935	.934
Q19_E					.818	.933	.932
Q20_E					.852	.934	.934
Q21_E					.839	.934	.933
Q22_E					.853	.935	.934
α with all	.778	.874	.820	.861	.866	.937	.937

Scale Cronbach's αs without each item

5. Confirmatory factor analysis

The items are divided by item type. There are arguments for doing exploratory or confirmatory.[1] In the paper We chose EFA based on wanting our procedure to parallel those of Ayers, Wright, and Thornton (2018). Two alternatives are a) to assume that there are four correlated factors for B–E for the sets of items, and fit these, and b) to assume the same model found in Ayers et al.. This will be done here. The packages **lavaan** (Rosseel, 2012) and **semPlot** (Epskamp, 2019) are used. Note that reading from `https` files at the moment works from Windows, but not other operating systems. The packages **RCurl** (Temple Lang, 2020) and **rio** (Chan, Chan, & Leeper, 2016) can be used in these circumstances (e.g., if using a Unix or Mac). The correlation table, made with the **xtable** package (Dahl, 2016), is shown in Table 1 (placed at the end of this document in order to fit . . . it is a sideways table).

```
library(lavaan)
# library(blavaan)    # If wishing to use a Bayesian approach
library(semPlot)
library(xtable)
#library(rio)         # use one of these (and import or getURL) if on a
#library(RCurl)       # non-Windows machine
forCFA <-
  read.csv("https://raw.github.com/dbrookswr/Psychometrics/master/PartnersBE.csv")
```

```
x <- cor(forCFA[,-1])
xtab <- matrix(sub("0.",".",sprintf("%2.3f",x)),nrow=nrow(x))
xtab[upper.tri(xtab,diag=TRUE)] <- NA
colnames(xtab) <- rownames(xtab) <- names(forCFA)[-1]
print(xtable(xtab[2:(nrow(xtab)),1:(ncol(xtab)-1)],caption="Correlation table.",
  label="tab:corr"),size="footnotesize",floating.environment='sidewaystable')
```

Here two confirmatory models will be examined. The first model is where each set of items B, C, D, and E, are influences by separate factors, and these factors are correlated. The second is the two-factor findings from Table 4 of Ayers et al. (2018).

```
model1 <-
 'Intrusion =~ Q3_B + Q4_B + Q5_B + Q6_B + Q7_B
  Avoidance =~ Q8_C + Q9_C
  NegMood =~ Q10_D + Q11_D + Q12_D + Q13_D + Q14_D + Q15_D + Q16_D
  HyperAr =~ Q17_E + Q18_E + Q19_E + Q20_E + Q21_E + Q22_E '
```

`Q10_D` did not load on either of the two factors in Ayers et al. (2018).

```
model2 <-
 'Birth =~ Q3_B + Q4_B + Q5_B + Q6_B + Q7_B + Q8_C + Q9_C + Q11_D + Q12_D
  General =~  Q13_D + Q14_D + Q15_D + Q16_D + Q17_E + Q18_E + Q19_E + Q20_E + Q21_E + Q22_E '
```

For comparability, here is model 1 without the tenth item.

[1] As well as for treating the responses as ordinal with models such as the partial credit model from IRT. In this paper and our original paper, the partial credit and EFA models were explored, yielded similar substantive findings, and we opted for EFA for communicative purposes.

```
model1a <-
 'Intrusion =~ Q3_B + Q4_B + Q5_B + Q6_B + Q7_B
  Avoidance =~ Q8_C + Q9_C
  NegMood =~ Q11_D + Q12_D + Q13_D + Q14_D + Q15_D + Q16_D
  HyperAr =~ Q17_E + Q18_E + Q19_E + Q20_E + Q21_E + Q22_E '
```

Confirmatory Factor Analysis

The models will be fit and a plot of the loading shown. These plots are close to the defaults, and can be improved. The statistical output is shown at the end.

```
fit1 <- cfa(model1, data = forCFA)
fit1a <- cfa(model1a, data = forCFA)
fit2 <- cfa(model2, data = forCFA)
```

Because these are based on different numbers of items comparisons should be made with caution, but it appears the general-birth symptom two-factor model of Ayers et al. (2018) fits similar tot he four-factor model. The BIC (Bayesian information criteria) are nearly equal. BIC prefers simpler models compared with more complex models, relative to the AIC (an information criterion), which shows support for the four model solution.

```
anova(fit1a,fit2)

## Chi-Squared Difference Test
##
##        Df   AIC   BIC  Chisq Chisq diff Df diff Pr(>Chisq)
## fit1a 146 13484 13647 535.68
## fit2  151 13505 13649 566.23     30.552       5  1.148e-05 ***
## ---
## Signif. codes:  0 '***' 0.001 '**' 0.01 '*' 0.05 '.' 0.1 ' ' 1
```

```
labels = c("Intrusions", "Avoidance",
            "Neg-Mood/Cog", "Hyperarousal")
semPaths(fit1,layout="circle",residuals=FALSE,nCharNodes = 0,
         what="std",sizeLat = 12)
```

```
#lavaanPlot(model = fit, covs = TRUE)
#lavaanPlot(model = fit, labels = labels, node_options =
#list(shape = "box", fontname = "Helvetica"),
#  edge_options = list(color = "grey"), coefs = TRUE, covs = TRUE)
```

Figure **??** shows that most of the loadings are as predicted. `Q10_D` doesn't have a large loading. This is not surprising as this had low correlations with all items (see Table 1). Its median correlation was: .173. Figure 2 has all the standardized loadings of a reasonable size.

```
semPaths(fit2,what="std",layout="circle",
         residuals=FALSE,nCharNodes = 0,sizeLat = 12)
```

The summary statistics for model 1 and model 2 are now provided for documentation purposes.

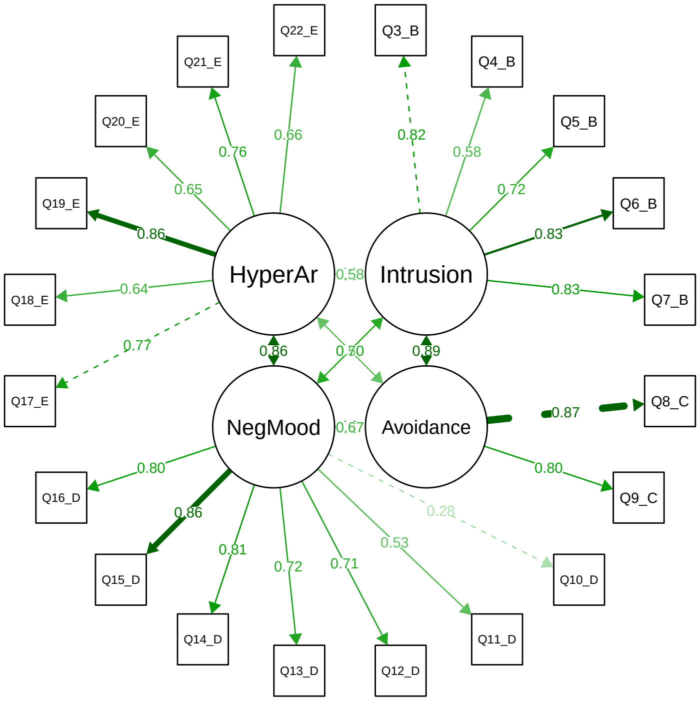

Figure 1: Path diagram for model 1, classical.

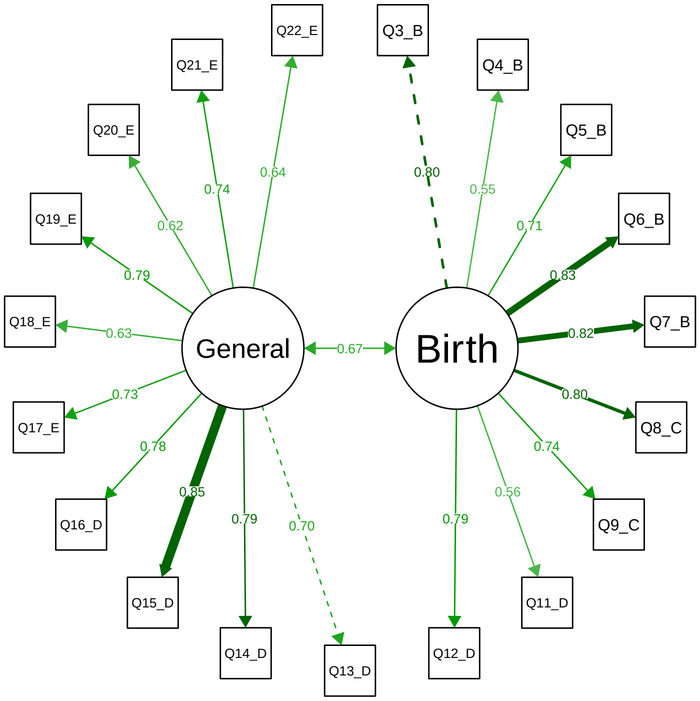

Figure 2: Path diagram for model 2, classical.

```r
summary(fit1, fit.measures=TRUE)
```

```
## lavaan 0.6-6 ended normally after 74 iterations
##
##   Estimator                                         ML
##   Optimization method                           NLMINB
##   Number of free parameters                         46
##
##   Number of observations                           301
##
## Model Test User Model:
##
##   Test statistic                               583.158
##   Degrees of freedom                               164
##   P-value (Chi-square)                           0.000
##
## Model Test Baseline Model:
##
##   Test statistic                              3853.975
##   Degrees of freedom                               190
##   P-value                                        0.000
##
## User Model versus Baseline Model:
##
##   Comparative Fit Index (CFI)                    0.886
##   Tucker-Lewis Index (TLI)                       0.867
##
## Loglikelihood and Information Criteria:
##
##   Loglikelihood user model (H0)              -6996.390
##   Loglikelihood unrestricted model (H1)      -6704.811
##
##   Akaike (AIC)                               14084.781
##   Bayesian (BIC)                             14255.308
##   Sample-size adjusted Bayesian (BIC)        14109.422
##
## Root Mean Square Error of Approximation:
##
##   RMSEA                                          0.092
##   90 Percent confidence interval - lower         0.084
##   90 Percent confidence interval - upper         0.100
##   P-value RMSEA <= 0.05                          0.000
##
## Standardized Root Mean Square Residual:
##
##   SRMR                                           0.064
##
## Parameter Estimates:
##
##   Standard errors                             Standard
##   Information                                 Expected
##   Information saturated (h1) model          Structured
##
## Latent Variables:
```

```
##                   Estimate  Std.Err  z-value  P(>|z|)
##   Intrusion =~
##     Q3_B              1.000
##     Q4_B              0.581    0.055   10.547    0.000
##     Q5_B              0.879    0.064   13.715    0.000
##     Q6_B              1.064    0.064   16.622    0.000
##     Q7_B              1.085    0.066   16.401    0.000
##   Avoidance =~
##     Q8_C              1.000
##     Q9_C              0.876    0.056   15.649    0.000
##   NegMood =~
##     Q10_D             1.000
##     Q11_D             2.517    0.580    4.339    0.000
##     Q12_D             4.014    0.874    4.592    0.000
##     Q13_D             4.085    0.888    4.598    0.000
##     Q14_D             4.976    1.066    4.669    0.000
##     Q15_D             5.298    1.127    4.702    0.000
##     Q16_D             4.770    1.023    4.663    0.000
##   HyperAr =~
##     Q17_E             1.000
##     Q18_E             0.662    0.059   11.246    0.000
##     Q19_E             1.062    0.068   15.551    0.000
##     Q20_E             0.698    0.061   11.366    0.000
##     Q21_E             0.986    0.072   13.605    0.000
##     Q22_E             0.858    0.074   11.644    0.000
##
## Covariances:
##                   Estimate  Std.Err  z-value  P(>|z|)
##   Intrusion ~~
##     Avoidance         0.723    0.076    9.549    0.000
##     NegMood           0.105    0.025    4.198    0.000
##     HyperAr           0.405    0.057    7.161    0.000
##   Avoidance ~~
##     NegMood           0.126    0.030    4.185    0.000
##     HyperAr           0.440    0.068    6.482    0.000
##   NegMood ~~
##     HyperAr           0.138    0.032    4.277    0.000
##
## Variances:
##                   Estimate  Std.Err  z-value  P(>|z|)
##    .Q3_B              0.328    0.033    9.896    0.000
##    .Q4_B              0.424    0.036   11.661    0.000
##    .Q5_B              0.460    0.042   10.977    0.000
##    .Q6_B              0.323    0.034    9.539    0.000
##    .Q7_B              0.358    0.037    9.707    0.000
##    .Q8_C              0.315    0.049    6.374    0.000
##    .Q9_C              0.454    0.049    9.236    0.000
##    .Q10_D             0.422    0.035   12.184    0.000
##    .Q11_D             0.561    0.047   11.866    0.000
##    .Q12_D             0.543    0.048   11.205    0.000
##    .Q13_D             0.540    0.048   11.160    0.000
##    .Q14_D             0.461    0.045   10.323    0.000
##    .Q15_D             0.343    0.037    9.285    0.000
```

```
##    .Q16_D             0.452    0.043   10.448    0.000
##    .Q17_E             0.522    0.050   10.427    0.000
##    .Q18_E             0.465    0.041   11.371    0.000
##    .Q19_E             0.310    0.035    8.745    0.000
##    .Q20_E             0.501    0.044   11.341    0.000
##    .Q21_E             0.530    0.050   10.504    0.000
##    .Q22_E             0.702    0.062   11.267    0.000
##     Intrusion         0.652    0.078    8.386    0.000
##     Avoidance         1.018    0.114    8.953    0.000
##     NegMood           0.035    0.015    2.343    0.019
##     HyperAr           0.751    0.098    7.641    0.000
```

```
summary(fit2, fit.measures=TRUE)
```

```
## lavaan 0.6-6 ended normally after 33 iterations
##
##   Estimator                                         ML
##   Optimization method                           NLMINB
##   Number of free parameters                         39
##
##   Number of observations                           301
##
## Model Test User Model:
##
##   Test statistic                               566.233
##   Degrees of freedom                               151
##   P-value (Chi-square)                           0.000
##
## Model Test Baseline Model:
##
##   Test statistic                              3784.697
##   Degrees of freedom                               171
##   P-value                                        0.000
##
## User Model versus Baseline Model:
##
##   Comparative Fit Index (CFI)                    0.885
##   Tucker-Lewis Index (TLI)                       0.870
##
## Loglikelihood and Information Criteria:
##
##   Loglikelihood user model (H0)              -6713.393
##   Loglikelihood unrestricted model (H1)      -6430.277
##
##   Akaike (AIC)                               13504.787
##   Bayesian (BIC)                             13649.364
##   Sample-size adjusted Bayesian (BIC)        13525.678
##
## Root Mean Square Error of Approximation:
##
##   RMSEA                                          0.096
##   90 Percent confidence interval - lower         0.087
##   90 Percent confidence interval - upper         0.104
##   P-value RMSEA <= 0.05                          0.000
```

```
##
## Standardized Root Mean Square Residual:
##
##   SRMR                                           0.058
##
## Parameter Estimates:
##
##   Standard errors                             Standard
##   Information                                 Expected
##   Information saturated (h1) model          Structured
##
## Latent Variables:
##                    Estimate  Std.Err  z-value  P(>|z|)
##   Birth =~
##     Q3_B              1.000
##     Q4_B              0.561    0.057    9.892    0.000
##     Q5_B              0.877    0.066   13.235    0.000
##     Q6_B              1.079    0.066   16.281    0.000
##     Q7_B              1.103    0.068   16.134    0.000
##     Q8_C              1.175    0.075   15.665    0.000
##     Q9_C              1.035    0.074   13.948    0.000
##     Q11_D             0.628    0.062   10.073    0.000
##     Q12_D             1.049    0.069   15.287    0.000
##   General =~
##     Q13_D             1.000
##     Q14_D             1.224    0.094   12.973    0.000
##     Q15_D             1.312    0.095   13.876    0.000
##     Q16_D             1.176    0.091   12.860    0.000
##     Q17_E             1.107    0.092   11.974    0.000
##     Q18_E             0.752    0.073   10.349    0.000
##     Q19_E             1.143    0.088   12.936    0.000
##     Q20_E             0.784    0.076   10.332    0.000
##     Q21_E             1.121    0.092   12.189    0.000
##     Q22_E             0.964    0.091   10.548    0.000
##
## Covariances:
##                    Estimate  Std.Err  z-value  P(>|z|)
##   Birth ~~
##     General           0.392    0.051    7.617    0.000
##
## Variances:
##                    Estimate  Std.Err  z-value  P(>|z|)
##    .Q3_B              0.355    0.033   10.627    0.000
##    .Q4_B              0.447    0.038   11.861    0.000
##    .Q5_B              0.483    0.043   11.345    0.000
##    .Q6_B              0.334    0.033   10.238    0.000
##    .Q7_B              0.365    0.035   10.322    0.000
##    .Q8_C              0.470    0.045   10.560    0.000
##    .Q9_C              0.566    0.051   11.170    0.000
##    .Q11_D             0.534    0.045   11.841    0.000
##    .Q12_D             0.414    0.039   10.725    0.000
##    .Q13_D             0.569    0.050   11.381    0.000
##    .Q14_D             0.496    0.046   10.736    0.000
```

```
##    .Q15_D              0.369    0.037    9.900    0.000
##    .Q16_D              0.480    0.044   10.809    0.000
##    .Q17_E              0.601    0.053   11.237    0.000
##    .Q18_E              0.483    0.041   11.678    0.000
##    .Q19_E              0.439    0.041   10.761    0.000
##    .Q20_E              0.529    0.045   11.681    0.000
##    .Q21_E              0.569    0.051   11.151    0.000
##    .Q22_E              0.744    0.064   11.638    0.000
##     Birth              0.625    0.076    8.194    0.000
##     General            0.549    0.081    6.809    0.000
```

References

Ayers, S., Wright, D. B., & Thornton, A. (2018). Development of a measure of postpartum ptsd: The city birth trauma scale. *Frontiers in Psychiatry*, *9*, 409. doi: 10.3389/fpsyt.2018.00409

Chan, C., Chan, G. C. H., & Leeper, T. J. (2016). **rio**: A Swiss-army knife for data file i/o [Computer software manual]. (R package version 0.3.0)

Dahl, D. B. (2016). **xtable**: Export tables to LaTeX or html [Computer software manual]. Retrieved from https://CRAN.R-project.org/package=xtable (R package version 1.8-2)

Epskamp, S. (2019). semPlot: Path diagrams and visual analysis of various sem packages' output [Computer software manual]. Retrieved from https://CRAN.R-project.org/package=semPlot (R package version 1.1.2)

Rosseel, Y. (2012). **lavaan**: An R package for structural equation modeling. *Journal of Statistical Software*, *48*(2), 1–36. Retrieved from http://www.jstatsoft.org/v48/i02/

Temple Lang, D. (2020). **RCurl**: General network (HTTP/FTP/...) client interface for R [Computer software manual]. Retrieved from https://CRAN.R-project.org/package=RCurl (R package version 1.98-1.2)

	Q3_B	Q4_B	Q5_B	Q6_B	Q7_B	Q8_C	Q9_C	Q10_D	Q11_D	Q12_D	Q13_D	Q14_D	Q15_D	Q16_D	Q17_E	Q18_E	Q19_E	Q20_E	Q21_E
Q4_B	.591																		
Q5_B	.692	.476																	
Q6_B	.634	.491	.600																
Q7_B	.622	.418	.540	.733															
Q8_C	.637	.326	.496	.649	.713														
Q9_C	.571	.378	.500	.602	.593	.695													
Q10_D	.083	.082	.168	.099	.089	.180	.173												
Q11_D	.464	.394	.429	.435	.394	.362	.390	.243											
Q12_D	.586	.322	.526	.656	.641	.666	.562	.258	.563										
Q13_D	.372	.362	.370	.413	.421	.388	.383	.205	.464	.543									
Q14_D	.419	.361	.318	.400	.443	.378	.390	.163	.406	.499	.607								
Q15_D	.475	.321	.338	.424	.486	.478	.400	.243	.367	.534	.591	.756							
Q16_D	.406	.273	.298	.417	.448	.461	.387	.190	.342	.550	.518	.656	.741						
Q17_E	.388	.237	.315	.349	.372	.357	.355	.142	.318	.458	.478	.533	.574	.528					
Q18_E	.321	.286	.248	.369	.368	.323	.245	.083	.319	.415	.453	.429	.491	.488	.550				
Q19_E	.384	.269	.309	.366	.415	.380	.302	.260	.298	.471	.523	.543	.615	.568	.712	.537			
Q20_E	.364	.369	.370	.354	.394	.309	.308	.198	.257	.396	.410	.409	.514	.440	.444	.512	.543		
Q21_E	.314	.322	.301	.314	.326	.280	.269	.221	.356	.404	.529	.582	.589	.561	.527	.401	.662	.495	
Q22_E	.299	.378	.316	.311	.332	.282	.304	.127	.375	.357	.437	.475	.496	.455	.459	.364	.565	.442	.579

Table 1: Correlation table.
